# A kingdom-specific protein domain HMM library for improved annotation of fungal genomes

**DOI:** 10.1186/1471-2164-8-97

**Published:** 2007-04-10

**Authors:** Intikhab Alam, Simon J Hubbard, Stephen G Oliver, Magnus Rattray

**Affiliations:** 1 School of Computer Science, University of Manchester, Kilburn Building, Oxford Road, Manchester M13 9PL, UK; 2 Faculty of Life Sciences, University of Manchester, The Michael Smith Building, Oxford Road, Manchester M13 9PT, UK

## Abstract

**Background:**

Pfam is a general-purpose database of protein domain alignments and profile Hidden Markov Models (HMMs), which is very popular for the annotation of sequence data produced by genome sequencing projects. Pfam provides models that are often very general in terms of the taxa that they cover and it has previously been suggested that such general models may lack some of the specificity or selectivity that would be provided by kingdom-specific models.

**Results:**

Here we present a general approach to create domain libraries of HMMs for sub-taxa of a kingdom. Taking fungal species as an example, we construct a domain library of HMMs (called Fungal Pfam or FPfam) using sequences from 30 genomes, consisting of 24 species from the ascomycetes group and two basidiomycetes, *Ustilago maydis*, a fungal pathogen of maize, and the white rot fungus *Phanerochaete chrysosporium*. In addition, we include the Microsporidion *Encephalitozoon cuniculi*, an obligate intracellular parasite, and two non-fungal species, the oomycetes *Phytophthora sojae *and *Phytophthora ramorum*, both plant pathogens. We evaluate the performance in terms of coverage against the original 30 genomes used in training FPfam and against five more recently sequenced fungal genomes that can be considered as an independent test set. We show that kingdom-specific models such as FPfam can find instances of both novel and well characterized domains, increases overall coverage and detects more domains per sequence with typically higher bitscores than Pfam for the same domain families. An evaluation of the effect of changing E-values on the coverage shows that the performance of FPfam is consistent over the range of E-values applied.

**Conclusion:**

Kingdom-specific models are shown to provide improved coverage. However, as the models become more specific, some sequences found by Pfam may be missed by the models in FPfam and some of the families represented in the test set are not present in FPfam. Therefore, we recommend that both general and specific libraries are used together for annotation and we find that a significant improvement in coverage is achieved by using both Pfam and FPfam.

## Background

The number of genomes being sequenced now exceeds 2000. Of these, as of February 2007, 510 are completed while 1091, 695 and 62 bacterial, eukaryotic and archaeal genomes (respectively) are still underway [1]. Much of this genomic sequence is relatively poorly annotated and one of the major challenges in bioinformatics is the computational annotation of this massive amount of data in a high-throughput manner [2]. Genome annotation can be classified into three levels: the nucleotide, protein and process levels [3]. Databases such as PROSITE [4], PRINTS [5], SMART [6], TIGRFAMs [7] or Pfam [8], which keep information in the form of motifs, alignment blocks, or profiles, provide a reference for the annotation at the protein level [9] where the main aim is to identify conserved regions and domains within the protein sequences predicted at the nucleotide annotation stage. InterPro [10] provides an integrated resource to cross-reference these motif or domain databases.

The Pfam database, in particular, has a wealth of information about approximately 8000 domains and plays a major role in achieving such high-throughput annotation of newly sequenced genomes, due to its specialized profile Hidden Markov Models (HMMs) [11,12]. TIGRFAMs is another similar database of protein families based on HMMs designed to specifically support large sequencing projects, although this has less coverage with under 2500 models in release 4.1, and is focused more towards complete proteins than domains. Profile HMMs are flexible, probabilistic models that can be used to describe the consensus patterns shared by sets of homologous protein/domain sequences. They summarise the shared statistical features of these homologous sequences in a way that allows efficient searching for matches in translated DNA sequences corresponding to predicted protein-coding genes. HMMs in the Pfam database are constructed from an alignment of a representative set of sequences for each protein domain, called a seed alignment. The seed alignments are tested and improved by manual curation, and by application to large databases like the Universal Protein (UniProt) database [13]. A key issue, though, is the trade-off between sensitivity and specificity of the representative seeds and the corresponding models. If the seeds get larger and increasingly general, then they may lose specificity.

It has previously been reported that more specific HMMs, built from sequences obtained from a less diverged set of species, can lead to improved sensitivity and specificity in the detection of domains and will therefore provide improved coverage when annotating proteins in related species [14]. The HMM library TLFAM-Pro has been developed for use with prokaryotes and some results of using the method have been described [15]. About 3000 ClustalW alignments from NCBI's database of Clusters of Orthologous Groups (COGs) [16], as of 2001, were used to compile HMMs. It was found that, although TLFAM-Pro demonstrated higher scores and longer alignments, a search of the test dataset against Pfam yielded more total hits, suggesting that TLFAM-Pro may provide a useful complementary resource to Pfam. This preliminary study was carried out in 2002, when both the number of domains in Pfam and the number of available genomes was much smaller than now and therefore it is unclear whether these results remain valid. It was also reported that archaeal- and fungal- specific TLFAM databases had been constructed, or were to be constructed in the near future, but we are not aware of any publications describing them and no implementation is currently available. In other restricted applications, it has been shown that kingdom-specific HMMs improve performance -, as shown for example, in the prediction of N-terminal myristoylation sites in plants [17]. However, as far as we are aware no large-scale study of the effectiveness of kingdom-specific HMMs for protein domain searching has been carried out. Given the rapidly increasing availability of un-annotated or partially annotated genomes across all kingdoms, it is important to determine whether more specific HMMs are useful for the annotation of these genomes. In this paper, we test this hypothesis specifically, taking the case of fungal genomes as an example.

A large number of complete and partial genome sequences have recently become publicly available for fungal species. We are involved in the development of the e-Fungi data warehouse, which provides tools for the comparative analysis of these genomes and associated functional data [18]. As part of this project we are developing a pipeline for the automated annotation of new genomes as they become available. We are therefore interested in developing methods for identifying protein domains and it is important to obtain the best coverage possible. In this paper we describe a fungal-specific HMM library that was developed to carry out this task. This serves as an example of a kingdom-specific HMM library, and we evaluate its performance in comparison to the more general Pfam database [19]. We compile the fungal-specific HMMs using genomic data from the 30 species represented in the current version of the e-Fungi data warehouse [18]. We evaluate the increase in coverage provided by the fungal-specific models over those 30 species. In order to test the method on previously unseen data, we then evaluate its performance on five more recently sequenced genomes that were not included in the first release of the e-Fungi database used to construct the models. Our results demonstrate that a fungal-specific library does provide a significant increase in coverage and that best performance is achieved by combining results from the kingdom-specific HMM library with results from the standard Pfam library. We investigate how this improved coverage affects the distribution of identified multi-domain proteins and we investigate the functional annotation of families that show the largest difference in performance between the two libraries.

## Results and discussion

### Comparison of FPfam and Pfam results for sequences from 30 fungal genomes

**Figure 1:**
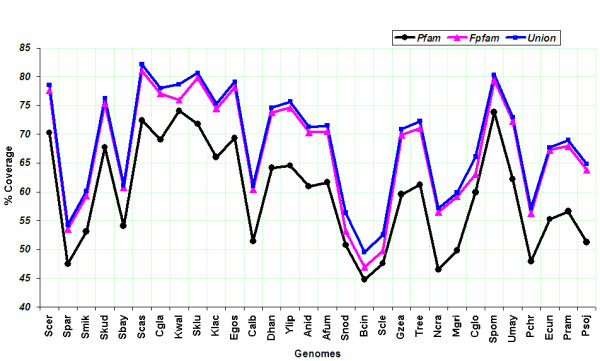
**Comparison of FPfam and Pfam results for sequences from the original 30 fungal genomes**. For each of the 30 original genomes, the Figure shows the percentage of sequences found to contain at least one domain using Pfam, FPfam and a combination. The average coverage was found to be 56.55% (Pfam), 64.29% (Fpfam) and 65.60% (combination). These matches were found against 2953 domains represented in the FPfam library. Please note that genome names are shown as a four letter code; comprising of the first letter from the genus name and 3 letters from the species name, also shown in the Table 1.

For each of the original 30 genomes (see Table 1) we calculated the percentage of sequences containing at least one domain using the two HMM libraries (see Figure 1). In this figure we only show result for the 2953 domains represented in this version of FPfam, since we are interested in comparing the sensitivity of the fungal-specific models compared to the general models for the same domains in Pfam. We found matches against these 2953 domains, with 56.55% average coverage of sequences in genomes by using Pfam, 64.29% by using FPfam, and 65.60% by combining them. Using FPfam, 15 genomes showed coverage of more than 70% of their sequences, while the other genomes had 46.99–69.89% of sequences covered. *Saccharomyces cerevisiae*, *Saccharomyces kudriavzevii*,*Saccharomyces castelli, Candida glabrata, Saccharomyces kluyveri*, *Eremothecium gossypii*, *Kluyveromyces waltii *and *Schizosaccharomyces pombe *achieved the highest coverage of above 75% of sequences. Coverage of sequences with domains using Pfam models is 2–13% lower than the coverage using FPfam models at the same E-value threshold. The combination of FPfam and Pfam improved the overall average coverage further. In addition to 151854 sequences commonly detected across all genomes, 24878 sequences were picked up using FPfam that were missed by Pfam, while only 3603 found with Pfam were missed by FPfam (for further details, see section on domain instances missed by Pfam below). These sequences could be added to the FPfam HMM seed alignments in order to improve coverage, but (in practice) both FPfam and Pfam will be used for annotation and it is therefore not necessary for FPfam to reproduce all Pfam hits.

### FPfam and Pfam results comparison for the test set of five fungal species

**Figure 2:**
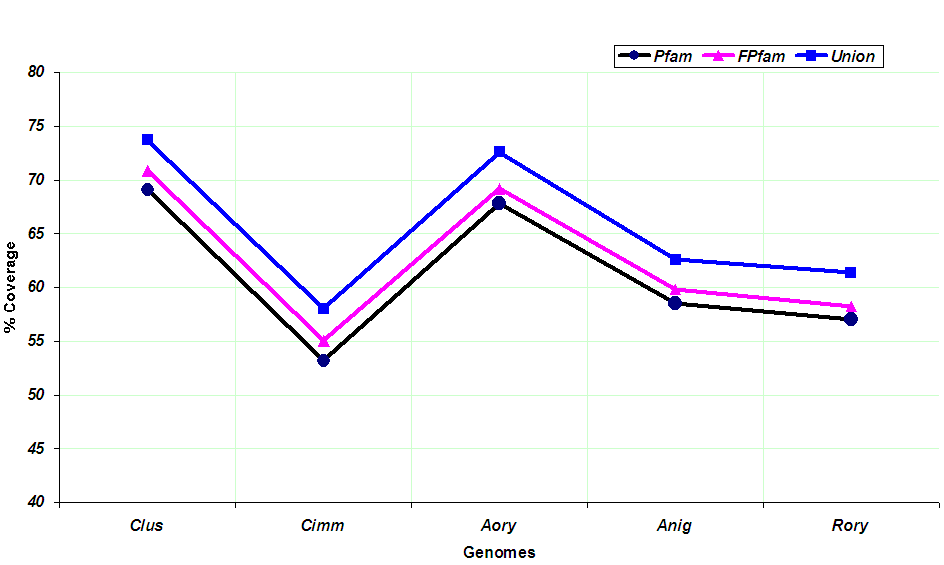
**Comparison of FPfam and Pfam results for sequences in the five new fungal genomes; the test case**. For each of the five fungal genomes, considered as a test case here, the Figure shows the percentage of sequences found to contain at least one domain using Pfam, FPfam and a combination. The average coverage was found to be 60.10% (Pfam), 61.53% (Fpfam) and 64.58% (combination) for the 2903 domains represented in the FPfam library. Please note that genome names are shown as a four letter code; comprising first letter from the species name and 3 letters from the genus, also shown in the Table 1

We have shown that the fungal-specific HMM library provides improved coverage over sequences within the original 30 genomes that were used to construct the library. Principally, however, we are interested in whether FPfam will be useful for searching new genomes that contain sequences not used to construct the library. A comparison of FPfam and Pfam results on the five new fungal genomes is shown in Figure 2. An average coverage of 60.10% and 61.53% was obtained using Pfam and FPfam, respectively; while combining the methods gives an improved coverage of 64.58%.

In addition to these results, Pfam also picked up some more domains that are not yet included in the FPfam libraries. This suggests that a further improvement could be obtained in the annotation of novel genomes by applying both general and species-specific domain libraries.

### Examples of domain instances missed by Pfam

The frequency or the number of domain instances recovered using Pfam and FPfam can be divided into two categories; *category A*, where both models identify domains and *category B*, where only one of the two models produce hits. Category A represents cases where both the libraries are broadly effective, while category B defines the libraries that are most effective in identifying additional domain instances. For clarity, the category B hits can further be divided into category B^f ^(FPfam alone) and category B^p ^(Pfam alone) hits. The number of domains and domain instances for category A, category B^f ^and category B^p ^in the training set of 30 and test set of five genomes are shown in Table 2 and Table 3. By looking at category B^f ^and category B^p^, in addition to category A hits, this shows clearly that the performance of FPfam is much better than Pfam, detecting both a higher number of domains and domain instances. This improved performance of the FPfam library is consistent across both the training and test set of genomes.

Going further, we considered the LICD family of proteins [PF04991] which are involved in phosphorylcholine metabolism [20]. From the Pfam database, available online [21], there are currently no hits for this family of proteins in fungal species. However, in this study, the original Pfam models and the FPfam models picked up 16 instances of category A hits. Furthermore, there are 53 instances of category B hits, where 51 were picked up by FPfam alone (category B^f ^hits) and 2 by Pfam alone (category B^p ^hits). Further examples of novel domains from the top category B hits, where there was no fungal hit previously known in the Pfam database, include the Laminin-B [PF00052] and Fascin [PF06268] domains. Interestingly, it has previously been reported that standard PFam HMMs are poor at distinguishing laminin domains compared to PANTHER [22]. Here, we note that the species-specific FPfam HMMs can indeed detect these domains with good sensitivity in fungal species. Another interesting example is Ribosomal_S6 [PF01250], a common and fundamental domain, currently assigned to 22 eukaryotic species by Pfam, only one of which is fungal. Here, FPfam is able to recover 26 B^f ^instances alone, no B^p ^hits were observed, while 13 Category A hits were found. This shows that the method is able to recover novel hits from both well-studied and rare domains, offering a similar sensitivity to alternative HMM building approaches [22] and extending the depth of annotation above that of the standard Pfam approach. More examples are shown in Table 4, where the top 20 domain families are sorted based on the fraction of category B^f ^hits compared to the B^p ^and category A hits. There are about 1400 domain families where the contribution of category B^f ^hits is at least 10% of the total, and this coverage goes up to at least 50% among 79 different families. It is due to these category B hits appearing in both columns (B^f ^and B^p^) that a combination of FPfam and Pfam results provides better coverage than either library by itself. The complete table for these results is shown in Additional File 1.

### Domains per sequence analysis

**Figure 3:**
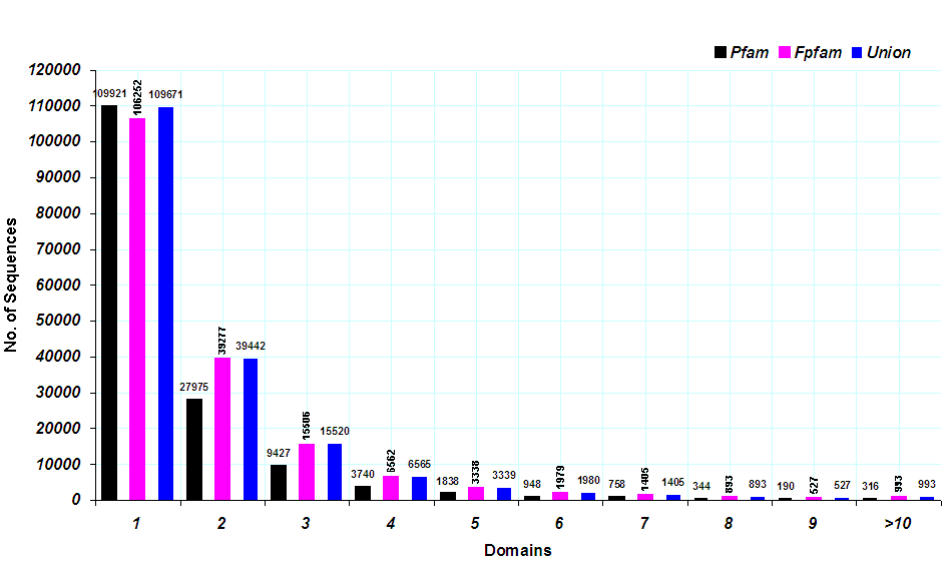
**Domains per sequence in the 30 original fungal genomes**. Domains per sequence, averaged over 30 original fungal genomes, are shown. The y-axis shows the number of sequences found with this number of domains. The FPfam library finds more sequences with more than one domain per sequence.

**Figure 4:**
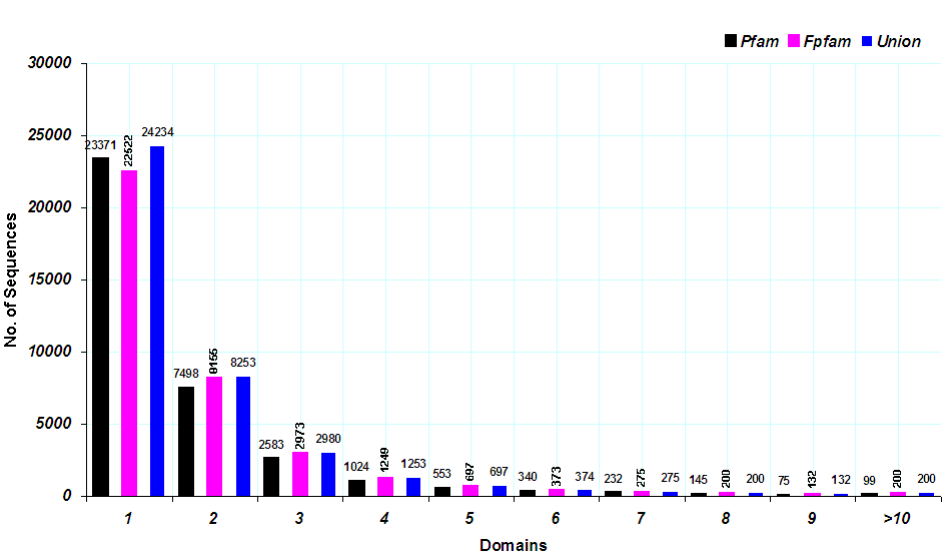
**Domains per sequence in the five new fungal genomes**. Domains per sequence, averaged over the five new fungal genomes used for testing, are shown. The y-axis shows the number of sequences found with this number of domains. The FPfam library finds more sequences with more than one domain per sequence.

To look at the coverage of domains in fungal sequences in a different way, the number of domains per sequence is presented in Figure 3 and Figure 4, averaged over the 30 original and five new fungal genomes, respectively. FPfam obtains less single-domain proteins and more multiple domain proteins than Pfam. It is clear from these figures that FPfam not only finds more proteins containing at least one domain but also unveils more domains per sequence.

### Comparison of bit-scores from Pfam and FPfam model searches

**Figure 5:**
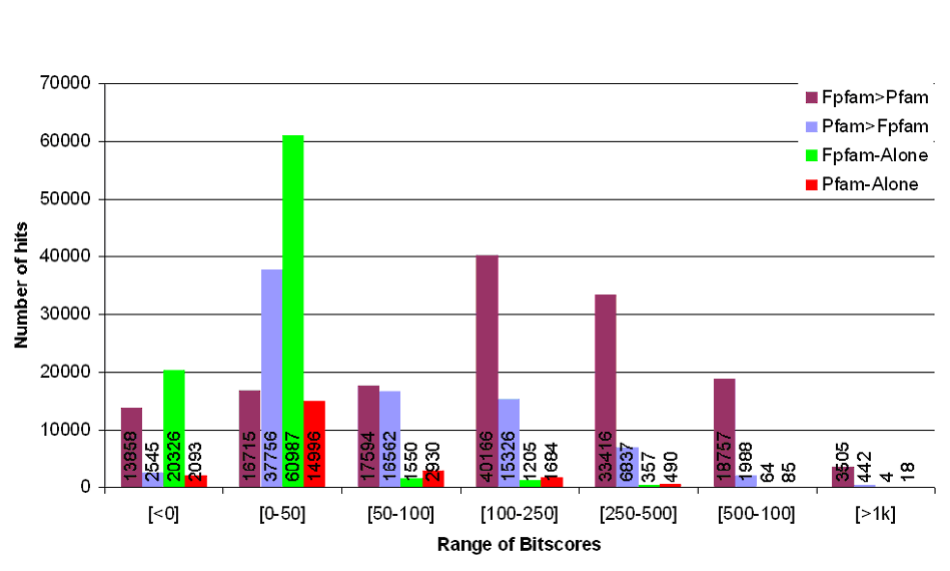
**Comparison of bitscore from Fpfam and Pfam HMM libraries in 30 genomes**. The X-axis shows different ranges of bitscores for which the frequency of FPfam>Pfam, Pfam>FPfam, no-Pfam and no-FPfam is calculated. To avoid frequencies being counted twice in cases where both Pfam and FPfam results are available, only the maximum score is assigned its respective bin.

**Figure 6:**
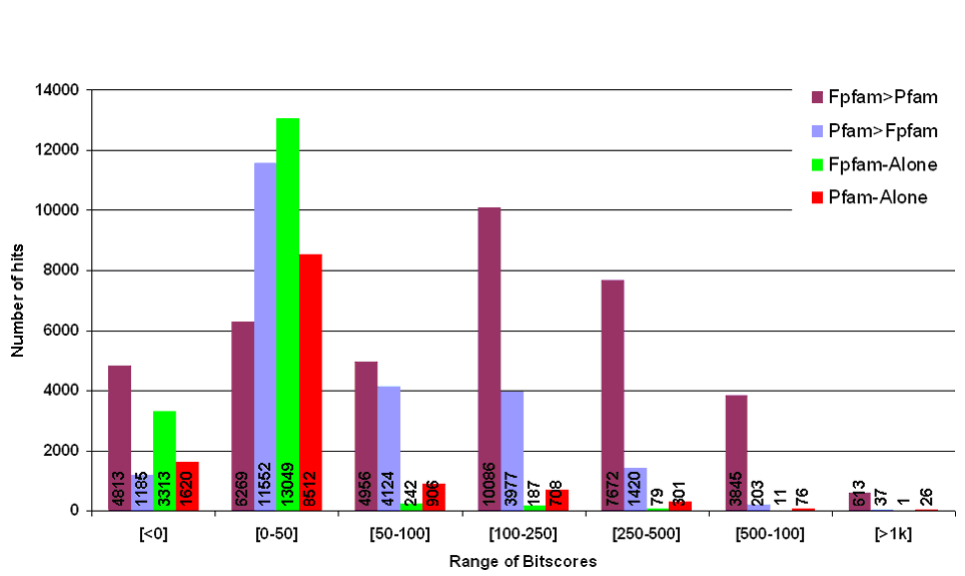
**Comparison of bitscore from Fpfam and Pfam HMM libraries in five genomes**. The X-axis shows different ranges of bitscores for which the frequency of FPfam>Pfam, Pfam>FPfam, no-Pfam and no-FPfam is calculated. To avoid frequencies being counted twice in cases where both Pfam and FPfam results are available, only the maximum score is assigned its respective bin. Generally, FPfam reports a higher bitscore.

In all of the analyses presented in this study we used the E-value as the only criterion to discriminate between true and false positives. By calibrating each library in the same way, these E-values should provide a similar false positive rate for each library and therefore make the results for each library comparable. However, it is also interesting to compare the distribution of bitscores on which these E-values are based, in order to identify any large differences between the corresponding models from each library. The bitscore is a normalized alignment score taking into account the underlying HMM scoring scheme, which is the same (in our case) for both models. To assess which of the two libraries produce a higher bitscore, histograms were constructed for the observed frequency of category A cases where bitscores for Pfam are higher than FPfam and *vice versa *(termed "Pfam>FPfam" and "FPfam >Pfam", respectively) and for the frequency of category B cases where either Pfam or FPfam results were available (termed "Pfam-alone" and "FPfam-alone"). The bitscores were placed in six bins of bitscore ranges. Only the maximum score from a pair was used to assign a hit to a bin when scores were available from both Pfam and FPfam, so each hit is counted only once. The histogram of frequencies for different ranges of bitscores from 30 fungal genomes is shown in Figure 5 and for five test genomes in Figure 6. From both Figures, it can be observed that for the higher bitscore ranges (>50) there are a larger number of cases where FPfam scores are greater than Pfam scores (see Fpfam>Pfam), while in the intermediate range (0 to 50) we see that although category A hits have larger Pfam scores on average, the number of cases found by Fpfam-alone is greatest in this range. In the lowest range (<0) we observe that for Category A hits FPfam also typically has higher bitscores. However, in this range we also see a relatively large number of cases found by Fpfam-alone in comparison to Pfam-alone.

### Effect of E-value cut-offs on sequence coverage

**Figure 7:**
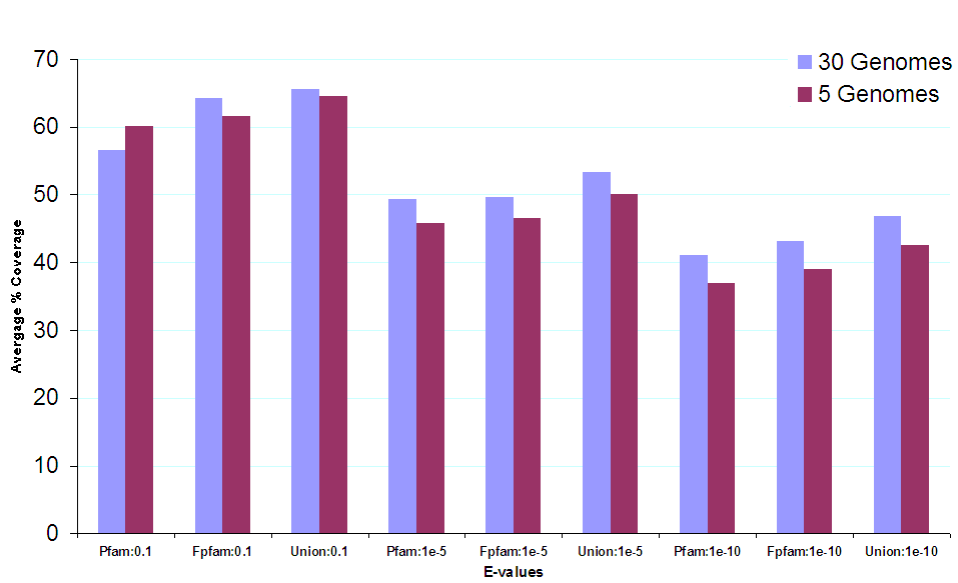
**Effect of different E-value cut-offs on sequence coverage**. The average percentage of sequences with at least one identified domain for the 30 original and five new fungal genomes is shown, for three different E-value cut-offs: 1e-1, 1e-5 and 1e-10. The percentage coverage using the FPfam library is higher than using Pfam alone. The best results are obtained when the outputs from the Pfam and FPfam library are combined.

To avoid any potential bias in the results due to selecting a single E-value cut-off to define hits, we reanalyzed the hmmpfam results using three different cut-offs, 1e-1, 1e-5 and 1e-10, as shown in Figure 7. The difference in results using the Pfam or FPfam libraries alone is most pronounced for the 30 fungal genomes that were used to train the FPfam library; while, for the five new genomes this difference is not as high (i.e. improved coverage of 1.43%, 0.79%, 1.96% for 1e-1, 1e-5, 1e-10, respectively). However, for the five test genomes if we look at the combination results they give (4.48%, 4.26%, 5.56% for 1e-1, 1e-5, 1e-10, respectively), i.e. significantly better coverage than using Pfam alone. This confirms that our fungal-specific HMM library produces many additional hits and suggests that the combination of the general Pfam library and a kingdom-specific library improves coverage, regardless of the E-value search sensitivity selected by the user.

## Conclusion

We have constructed a fungal-specific HMM library, FPfam, using sequences from 30 genomes and tested its performance against sequences from five new genomes. Our results show that FPfam provides improved sensitivity and coverage for domains represented in the library. By using FPfam, more sequences can be annotated as containing at least one of these domains and more multi-domain proteins are found at a given E-value cut-off. The best performance is obtained by combining FPfam with the general-purpose Pfam library, which finds some sequences missed by FPfam and allows additional domains to be located that are not represented in the current version of the FPfam library. Use of a kingdom-specific HMM library therefore effectively reduces the "twilight" zone and finds a significant number of difficult cases that might otherwise be missed. Indeed, the method demonstrates the ability to annotate additional examples of otherwise well-characterised, ubiquitous domains that Pfam and fungal-specific, rare motifs that are generally not well represented in the standard PFam HMM library.

Currently we are applying the domainer/mkdom algorithms [23] for all predicted proteins from the 35 fungal species, in order to have a database like Pfam-B providing coverage for all protein sequences in our e-Fungi fungal database. The FPfam libraries will then be used in order to classify all fungal sequences into super-families, families and subfamilies in a hierarchical fashion. The FPfam families will be made available as full alignments of these domains.

## Methods

### The Pfam database

Pfam is a database of multiple alignments of conserved regions or domains in proteins. Current release 18 of Pfam comprises alignments for more than 7973 domains [8]. The Pfam database has two parts: Pfam-A contains models constructed from human-curated multiple alignments covering 75% of UniProt [24] (the largest available collection of protein sequences), while Pfam-B has models constructed from alignments obtained by an automated clustering of the rest of UniProt derived from the Prodom database [25]. A recent development in the Pfam infrastructure is called Pfam clans or Pfam-C; this contains information about Pfam families that arise from a common ancestor. With ever-increasing coverage in protein databases, and based on human curated alignments, Pfam is a highly suitable and useable database for the large-scale annotation of proteins arriving from newly sequenced genomes. The easiest way to do this is to scan newly predicted Open Reading Frames (ORFs) against the HMMs using hmmpfam, provided in the HMMER package [26].

A typical Pfam-A entry contains a seed alignment, an alignment of a representative set of sequences, an HMM built using the seed alignment, a full alignment of all (detectable) sequences in the family and a description of the family with additional details such as the threshold parameters used to create the full alignment. Pfam seed alignments are saved and remain stable as long as they are able to detect all the known members of the family; otherwise the missing members are added to the alignment to improve the sensitivity of the HMMs. Seed and full alignments are curated manually and then the Pfam-A entry is annotated and linked to other motif databases [19].

### Identifying Pfam domains in 30 fungal species

Predicted ORFs from 30 fungal genomes, including two oomycetes, were obtained from the Broad Institute. These sequences were filtered for a length of more than 40 amino acids and the resulting proteome sizes for each genome are shown in Table 1. Pfam database release 18 was downloaded and installed locally. Each fungal sequence was scanned against Pfam HMMs using hmmpfam, from the HMMER package, applying an E-value cut-off of 0.1. With this cut-off, 57.15% of the total fungal proteins were found to contain at least one Pfam domain and 5314 different Pfam domains were detected in these 30 fungal species.

### Constructing a fungal-specific HMM library (FPfam)

We adopt the following procedure to construct a fungal-specific HMM library from the 30 original genomes:

a. For each domain, a maximum of two protein sequences per genome below an E-value cut-off of 1e-3 were obtained from the training dataset of fungal genomes. The training set of genomes is shown without asterisks in both Table 1 and the fungal species tree [see Additional File 2]. To avoid any bias towards the more closely related set of five genomes from *Saccharomyces *'*sensu stricto' *clade, the number of sequences to be included in the seed alignment from this group was reduced to a maximum of six. The E-value of 1e-3 was used to reduce the probability of introducing false positive hits into the seed alignments. A restriction of at least five sequences per model with an E-value less then 1e-3 reduced the number of domains to 2953. Furthermore, to avoid models becoming too specific, a maximum of four sequences were added from representative species of the different domains of life, selecting one homologue from Human, Mouse, plants and bacteria where available.

b. The set of sequences gathered for each of the 2953 domains was aligned using ClustalW [27]. To be compatible with Pfam, the alignment format was converted to selex.

c. All domain alignments were gathered into a single flatfile, adding the default Pfam-A annotation and parameters.

d. Global and local HMMs were constructed using hmmbuild from HMMER.

e. HMMs were calibrated using hmmcalibrate from HMMER.

f. The resulting fungal specific Pfam-A like database, from now on called FPfam, was indexed for sequence comparison using hmmpfam.

Protein sequences from 30 fungal genomes were scanned through the fungal version of Pfam (FPfam) database with the E-value cut-off of 0.1. FPfam results were compared with those obtained from searches against Pfam HMMs using the same E-value cut-off.

### Testing FPfam on five new genomes

As a test case, ORFs from five more recently sequenced fungal genomes were obtained from the Broad Institute [28] and from the DSM [29]. These are the species marked with asterisks in Table 1 and the phylogenetic tree [see Additional File 2]. These genomes were filtered removing protein sequences with lengths less than 40 amino acids. The resulting size of the proteome for each of the five new fungal genomes used in this test is shown in Table 1.

To perform the Pfam and FPfam comparison, each sequence from the five new fungal genomes was scanned against the HMMs from both libraries, using hmmpfam. The same E-value cut-off of 0.1 was applied in both cases. The libraries are calibrated in the same way, so we expect that the same E-value will result in a similar number of false positives in each case.

### Comparison of bitscores between FPfam and Pfam hits

After the completion of all the hmmpfam searches against the training and test set of genomes, using both the Pfam and FPfam HMMs, the hmmer normalized alignment scores (known as bitscores) were extracted. We divided the results into two main categories: A, where hits were available from both the Pfam and FPfam libraries and B, where one of the libraries did not produce any hits. Bitscores were assigned to six bins of bitscore ranges and the frequency of hits calculated for category A, where the FPfam score is higher than Pfam and *vice versa *(named Pfam>FPfam and FPfam>Pfam respectively) and for category B where either FPfam or Pfam results (named Pfam-alone and FPfam-alone), respectively, were missing. To avoid frequencies being counted twice for category A where both Pfam and Fpfam bitscores were available, only the maximum score of the two was used to determine its respective bin.

### Effect on the coverage of domains by varying E-value thresholds

The probability of false positives when searching a database of sequences is expressed in terms of E-values. To test the effect of E-value changes, we compared the coverage of sequences with at least one domain detected by either FPfam or Pfam alone to that of domains detected by considering the results from both libraries, applying a range of different E-value cut-offs (0.1, 1e-5, 1e-10).

## Authors' contributions

IA carried out the analysis and drafted the manuscript. SJH participated in the design of the study, interpretation of the results and manuscript preparation. SGO participated in the design of the study and manuscript preparation. MR coordinated the study, participated in the design and helped to draft the manuscript. All authors read and approved the final manuscript.

## Supplementary Material

Additional file 1 All detected domain families and the respective number of hits against Pfam and FPfam. A table showing frequencies of all the domains detected by both Pfam and FPfam (category A hits) or by individual libraries (category B hits), sorted based on category B^f ^(FPfam alone) hits. Click here for file

Additional file 2 Phylogenetic analysis of 35 fungal species. Phylogenetic tree relating the 35 genomes used in the study and description of methods used to construct the tree. Click here for file

## Figures and Tables

**Table 1:** Proteome sizes of 30 original fungal genomes and five test genomes (shown by asterisks)

**i**	**4-letter code**	**Genome**	**Sequences**
1	Scer	*Saccharomyces cerevisiae*	5823
2	Spar	*Saccharomyces paradoxus*	8564
3	Smik	*Saccharomyces mikatae*	11731
4	Skud	*Saccharomyces kudriavzevii*	3766
5	Sbay	*Saccharomyces bayanus*	13975
6	Scas	*Saccharomyces castellii*	4674
7	Sglab	*Candida glabrata*	5192
8	Kwal	*Kluyveromyces waltii*	5205
9	Sklu	*Saccharomyces kluyveri*	2963
10	Egos	*Eremothecium gossypii*	4723
11	Klac	*Kluyveromyces lactis*	5335
12	Calb	*Candida albicans*	14217
13	Dhen	*Debaryomyces hansenii*	6274
14	Clus	Candida lusitaniae*	5940
15	Ylip	*Yarrowia lipolytica*	6531
16	Cimm	Coccidioides immitis*	5940
17	Anid	*Aspergillus nidulans*	9523
18	Afum	*Aspergillus fumigatus*	9926
19	Aory	Aspergillus oryzae*	12062
20	Anig	Aspergillus niger*	14090
21	Snod	*Stagonospora nodorum*	16312
22	Bcin	*Botrytis cinerea*	9634
23	Sscl	*Sclerotinia sclerotiorum*	14145
24	Gzea	*Gibberella zeae*	11633
25	Tree	*Trichoderma reesei*	9783
26	Ncra	*Neurospora crassa*	9794
27	Mgri	*Magnaporthe grisea*	11082
28	Cglob	*Chaetomium globosum*	11046
29	Spom	*Schizosaccharomyces pombe*	4993
30	Umay	*Ustilago maydis*	6519
31	Pchr	*Phanerochaete chrysosporium*	10915
32	Rory	Rhizopus oryzae*	17298
33	Ecun	*Encephalitozoon cuniculi*	1996
34	Pram	*Phytophthora ramorum*	15876
35	Psoj	*Phytophthora sojae*	18986

**Table 2:** The number of instances for category A, B^f^**and B**^p^

**No of Instances:**	**30 Genomes**	**5 Genomes**
Category A	324758	67645
Category B^f^	38075	5079
Category B^p^	3814	1951

**Table 3:** The number of domains for category A, B^f^**and B**^p^

**No of Domains:**	**30 Genomes**	**5 Genomes**
Category A	2953	2839
Category B^f^	2749	1314
Category B^p^	760	676

**Table 4:** Category-A and B instances for FPfam and Pfam domains in 30 original and five test genomes

**Domain**	**Description**		**Total**	**Category B (f)**		**Category B (p)**		**Category A**	
		**B**^**f**^**frac**	**B**^**f**^**: B**^**p**^**:A**	**FPfam30**	**FPfam5**	**Pfam30**	**Pfam5**	**FP:Pf30**	**FP:Pf5**
DUF229	Protein of unknown function (DUF229)	87.5	7:0:1	6	1	0	0	1	0
LicD	LICD Protein Family	73.91	51:2:16	51	0	2	0	9	7
Neugrin	Neugrin	71.43	45:0:18	41	4	0	0	11	7
Copper-bind	Copper binding proteins, plastocyanin/az	70.59	36:1:14	32	4	1	0	12	2
DUF946	Plant protein of unknown function (DUF94	68.33	41:3:16	35	6	1	2	12	4
DUF143	Domain of unknown function DUF143	67.44	29:0:14	27	2	0	0	8	6
Laminin_B	Laminin B (Domain IV)	66.67	2:0:1	2	0	0	0	1	0
Ribosomal_S6	Ribosomal protein S6	66.67	26:0:13	26	0	0	0	6	7
Fascin	Fascin domain	66.67	6:0:3	6	0	0	0	2	1
Fungal_ODC_AZ	Fungal ornithine decarboxylase antizyme	66.67	8:0:4	6	2	0	0	4	0
Chitin_bind_3	Chitin-binding domain	65.04	80:5:38	80	0	3	2	35	3
DUF1279	Protein of unknown function (DUF1279)	64.81	35:0:19	34	1	0	0	11	8
GCC2_GCC3	GCC2 and GCC3	64.29	27:0:15	26	1	0	0	15	0
TRI5	Trichodiene synthase (TRI5)	64	16:0:9	14	2	0	0	5	4
Hormone_1	Somatotropin hormone family	63.64	14:0:8	13	1	0	0	5	3
Sulfotransfer_1	Sulphotransferase domain	63.64	7:1:3	7	0	1	0	3	0
Far-17a_AIG1	FAR-17a/AIG1-like protein.	62.5	40:0:24	36	4	0	0	18	6
UPF0139	Uncharacterised protein family (UPF0139)	61.9	13:0:8	12	1	0	0	5	3
ATP-synt_E	ATP synthase E chain	61.7	29:0:18	26	3	0	0	15	3
LRRNT	Leucine-rich repeat N-terminal domain	61.54	8:1:4	7	1	1	0	4	0
