# Supplementary material for: A kingdom-specific protein domain HMM library for improved annotation of fungal genomes
Source: BMC Genomics. 2007 Apr 10;8:97. doi: 10.1186/1471-2164-8-97 (PMC1854895; doi:10.1186/1471-2164-8-97)
Supplement: Additional file 2 — Phylogenetic analysis of 35 fungal species. Phylogenetic tree relating the 35 genomes used in the study and description of methods used to construct the tree. [file 1471-2164-8-97-S2.doc]

Additional File 2

Phylogenetic analysis of 35 fungal species:


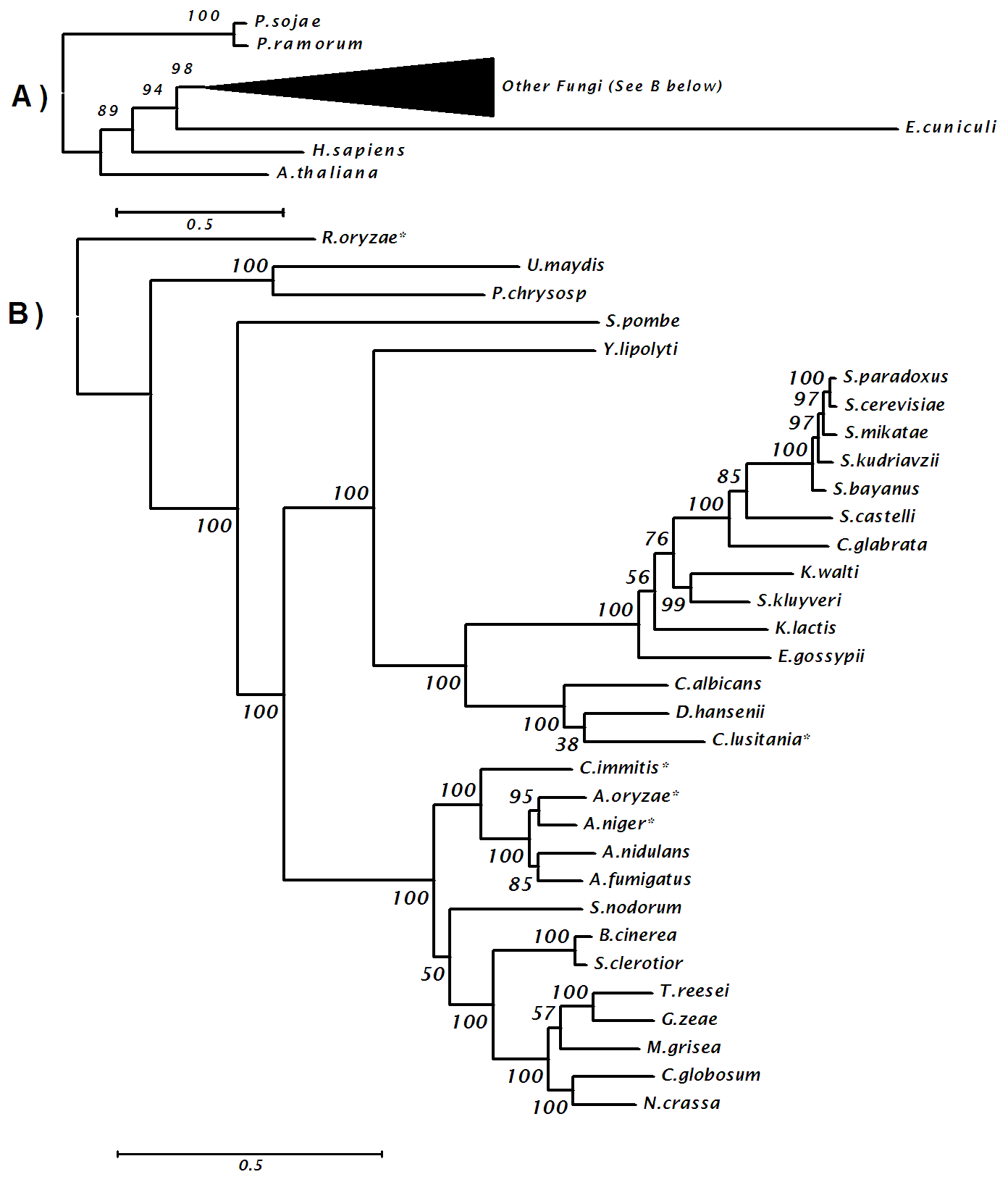


Species trees based on concatenated proteins from several universal families for which orthology can be unambiguously determined. The first tree (a), only part of which is shown, was constructed using 12 universal protein families taken from all the fungal species studied in this paper and including two oomycetes, *Phytophthora ramorum* and *Phytophthora sojae*, as well as *Homo sapiens* and *Arobidopsis thaliana* included to root the fungal tree. The second tree (b) is based on 29 universal protein families taken from 32 fungal species (excluding *Encephalitozoon cuniculi* and the two oomycetes). The five species marked with an asterisk are the new genomes used for testing the FPfam library.

Phylogenetic analysis of 35 fungal species

***Methods***

The open reading frames (ORFs) for 35 fungal genomes (including the 30 used to build FPfam and 5 used to test FPfam) were downloaded from the Joint Genome Institute (JGI), Japan and the Broad Institute of MIT, USA websites. The following procedure was used to construct a species tree for 35 fungal genomes.

1. Two sets of protein family data were prepared by selecting only sequences with unambiguous orthologues in all species under consideration. Orthology was established by choosing universal protein families (UPFs, i.e. those with at least one member in all taxa under consideration) and by inspecting rough gene trees, constructed by neighbour joining [1], to determine whether the order of duplication events was unambiguous. The smallest UPFs were considered first since these have the least duplications and arrangements are therefore easier to resolve in most cases. By this method we obtained:
   1. 12 UPFs present across the 35 taxa, *Homo sapiens* and *Arabidopsis thaliana* genomes.
   2. 29 UPFs present across these 35 fungal genomes.
2. All sequences comprising each UPF were aligned, deleting columns from the alignment with more than 50% gaps.
3. For each dataset, a and b, proteins from all UPF alignments were concatenated in order to have one continuous alignment for each genome.
4. PhyML [2], a maximum likelihood tree construction method, was applied using a JTT model of substitution [3] with a Gamma+Invariant variable rates model with four rate categories [4] for both dataset a and b. Trees were built using 100 bootstrap replicates to assess confidence in the inferred clade membership. The likelihood of all topologies obtained was checked for each bootstrap sample in order to reduce the probability of obtaining a local, rather than global, optimum of the likelihood.

**REFERENCES**

1. Saitou N, Nei M: **The Neighbor-Joining Method - a New Method for Reconstructing Phylogenetic Trees**. *Molecular Biology and Evolution* 1987, **4**(4):406-425.

2. Guindon S, Gascuel O: **A simple, fast, and accurate algorithm to estimate large phylogenies by maximum likelihood**. *Syst Biol* 2003, **52**(5):696-704.

3. Jones DT, Taylor WR, Thornton JM: **The Rapid Generation of Mutation Data Matrices from Protein Sequences**. *Computer Applications in the Biosciences* 1992, **8**(3):275-282.

4. Yang ZH: **Maximum-Likelihood Phylogenetic Estimation from DNA-Sequences with Variable Rates over Sites - Approximate Methods**. *Journal of Molecular Evolution* 1994, **39**(3):306-314.
